# Supplementary material for: Expression Patterns of Drosophila Melanogaster Glutathione Transferases
Source: Insects. 2022 Jul 7;13(7):612. doi: 10.3390/insects13070612 (PMC9318439; doi:10.3390/insects13070612)
Supplement: Supplementary file 1 [file insects-13-00612-s001.zip › insects-1788828-supplementary.pdf]

Table S1. List of the primers used for qRT-PCR analysis.

CG	Gene Name	Forward primer Sequence	Reverse primer sequence	Efficiency (%)
CG10045	GST D1	GTACAGGGAGTCGGTCTTGC	GCTGGTGGACAACGGATT	103
CG4181	GST D2	TGCTGAAGTCGAACTCACTAACTT	CGGACATTGCCATCCTGT	108,4
CG4381	GST D3	AATCGAAAGTCTCCTGGACCT	TTTACCACCGGTCAGTTTGG	105,5
CG11512	GST D4	TCAATCCCCAGCACACCATTCC	CGTCCTTGCCGTACTTTTCCAC	105,4
CG12242	GST D5	TCAAAGATTTCGAAAGTGGAAAC	CCACCTCACAGTGGCTGATA	106,7
CG4423	GST D6	CCATCCAGGAAGTTGTTGAGA	ACCCGGAACTCAGGAGAATC	98,3
CG4371	GST D7	AATGGTGTGCTGTGGGTTAAT	CAACACGATGGAGGGTGAC	110,6
CG4421	GST D8	CAATCCACAGCACTGCATTCCC	AGTCATCAGCGCCGTACTTCTC	83,5
CG10091	GST D9	TCGAGGATGTGAAGAAGCCAGC	AGAAGCGCAAAATCGGCCAG	92,4
CG18548	GST D10	CAACCAGCCCAGTTTTCCT	TGGCACGTTGGTACGAGA	108,1
CG17639	GST D11	ACTTCACCATCGCGGATCTCAC	GGCCTTGAACATATCGGCCAAC	104,5
CG5164	GST E1	TCACATATTCCTCGCTCAGGT	GGACTACGAGTACAAGGAGGTGA	95,9
CG17523	GST E2	TGGCCATATGCATCTTTAATGT	TCGCCAAGTAAGCCTGGTA	109,3
CG17524	GST E3	ACCCCAAGGATCTGAAGAAGCG	TGGCATCTACAGGCAGCAACAC	105,4
CG17525	GST E4	AAAGTACGCGCCAAGCGATG	AGGGCAGACTCGAAGATGACAG	107
CG17527	GST E5	CGAGTCCCAAATGTAGTTACCA	AAAAAGAATCCAGAGCACACG	94,7
CG17530	GST E6	CATGCGAATCCCAGATGTAG	AGAAGAATCCACAGCATACGG	95,7
CG17531	GST E7	CGACGTAGTCATTTCCAGCA	GCGTTACGATGCGATTATTG	109
CG17533	GST E8	CTTGGGAATCGTCGTCTGA	GGTCTTTGTCAACGGACTGAG	96,7
CG17534	GST E9	GCGTGACTCTCCCAGATGA	AAGAATCCGCAGCACACG	104,3
CG17522	GST E10	ACGTATCTCCGCATTGCCCTTC	TCACTGTTTTCCAGCTCCGCTC	93
CG5224	GST E11	CTTGTCGCCCACCAAGTAA	AGAAGGCCTATGATGGCTTG	107,3
CG16936	GST E12	TTCCGGAGTCAGATGCTCTC	GATCGGACTCGACCTTGAAG	107,8
CG11784	GST E13	TGGGGGTTTAGCTTGACAAA	CCTGGTGGCCAAACTTATTG	108,4
CG4688	GST E14	TGTGGCACATCATGAACGCAAG	TACCGCTGATGACGATGGGAAC	109,2
CG15693	RPS20	GACGATCTCAGAGGGCGAGT	TGTGGTGAGGGTTCCAAGAC	95,3
CG8615	RPL18	TGCTGGCACTCAGGATGGTT	GCAAGCCAGCACTGAATACG	102,7
CG7939	RPL32	AGCTTCAAGATGACCATCCGCC	TGCGCTTGTTCGATCCGTAACC	98,6
